# The Role of Health Literacy in Explaining the Relation between Educational Level and Decision Making about Colorectal Cancer Screening

**DOI:** 10.3390/ijerph16234644

**Published:** 2019-11-22

**Authors:** Anke J. Woudstra, Ellen M. A. Smets, Mathilde G. E. Verdam, Mirjam P. Fransen

**Affiliations:** 1 Department of Public Health, Amsterdam Public Health Research Institute, Amsterdam UMC, University of Amsterdam, Meibergdreef 9, 1105 AZ Amsterdam, The Netherlands; m.p.fransen@amsterdamumc.nl; 2 Department of Medical Psychology, Amsterdam Public Health Research Institute, Amsterdam UMC, University of Amsterdam, Meibergdreef 9, 1105 AZ Amsterdam, The Netherlands; e.m.smets@amsterdamumc.nl (E.M.A.S.); m.g.e.verdam@amsterdamumc.nl (M.G.E.V.); 3 Department of Methodology and Statistics, Institute of Psychology, Leiden University, Wassenaarseweg 52, 2333 AK Leiden, The Netherlands

**Keywords:** education, health literacy, informed decision making, colorectal cancer screening, mediation analysis, structural equation modelling

## Abstract

Little is known about why educational inequalities exist in informed decision making in colorectal cancer (CRC) screening. Insight into the role and impact of health literacy is essential for intervention development. This study investigates associations between health literacy and informed decision making in CRC screening and explores to what extent health literacy mediates the association between education and informed decision making in CRC screening. In total, 696 individuals eligible for CRC screening (55–75 years of age) were recruited from online panels and filled in an online questionnaire at T0 (*n* = 696), T1 (*n* = 407) and T2 (*n* = 327). A hypothetical mediation model was tested using structural equation modelling. Outcomes included CRC knowledge, CRC screening knowledge, attitude, injunctive norm, descriptive norm, risk perception, self-efficacy, decisional conflict and decisional certainty. Health literacy domains included *Comprehension*, *Application*, *Numeracy* and *Communication*. *Comprehension*, *Application* and *Numeracy*, were found to mediate the association between education and knowledge about CRC and CRC screening, injunctive norm, descriptive norm, decisional conflict and decisional certainty. In light of these findings, targeting multiple health literacy domains in decision-support interventions is essential for facilitating informed decision making in CRC screening.

## 1. Introduction

Colorectal cancer screening (CRC) is an effective strategy to reduce the mortality and morbidity in the population [1]. However, as with all screening programs, not all participants will benefit from participation, and screening programs have the potential to do harm through risks of the procedure itself, false test results, unnecessary detection and anxiety [2,3]. It is increasingly being recognized that screening programs should therefore aim to facilitate informed decision making (IDM) [4]. IDM refers to the process of weighing up individual potential benefits and harms of CRC screening [4]. However, IDM in CRC screening is difficult to achieve, especially for those with a lower educational level. Previous studies found that individuals with a lower educational level have lower knowledge on cancer, cancer risk and cancer screening and are less likely to make informed decisions [5,6].

Health literacy (HL) may be a possible mediator in the relation between educational level and IDM in CRC screening. HL is generally defined as the ability to access, understand, appraise and apply information in order to make informed health decisions [7]. Low HL has been shown to be a key determinant of health and a stronger predictor of an individual’s health status than educational level [8]. Low HL is more prevalent among those with a low educational level. However, individuals with a higher educational level may also have low HL [9]. Although HL has been increasingly considered as the missing link or mediating variable in inequalities in health and use of health care [3], it has hardly been empirically investigated in cancer screening.

HL has shown to be associated with elements of IDM, although the evidence is scarce and contradictory [10]. For instance, several studies found that individuals with a lower HL have lower CRC screening knowledge [11,12]. However, other studies found no association between HL and CRC screening knowledge [13,14]. To date, most existing studies on HL and decision making are limited by (1) the assessment of merely functional HL (i.e., basic reading and writing skills) and (2) the assessment of single elements (e.g., knowledge, attitude-uptake consistency or deliberation) with regard to IDM in CRC screening [10].

For optimizing decision-support interventions, more insight is needed into the role of HL in educational inequalities in IDM in CRC screening. Insight into how and which HL skills play a role in decision making enables the development of tailored communication and decision-support tools, and the evaluation of such interventions. It is therefore crucial to apply comprehensive HL measures in this particular context [15]. We previously underlined the importance of assessing multiple domains of HL (*Comprehension*, *Appraisal*, *Application*, *Numeracy* and *Communication*) in the context of IDM in CRC screening. IDM about CRC screening not only requires the ability to read and understand CRC screening information, individuals also need the ability to judge, discuss and apply information for personal relevance [15,16].

**Figure 1:**
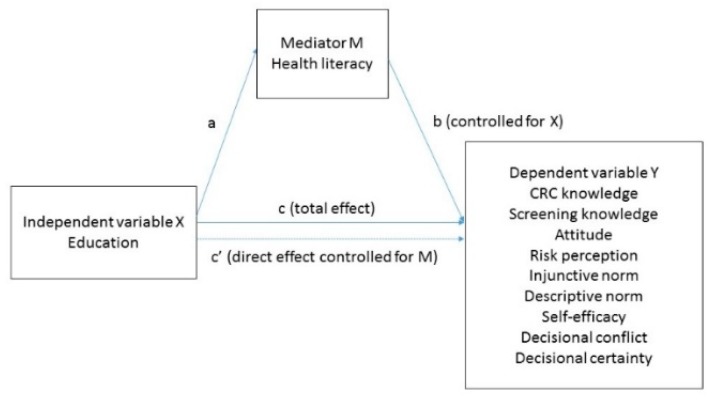
Hypothesized conceptual model: The effects of education on informed decision making (IDM) are (partly) mediated by domains of health literacy (HL).

As individuals with a lower educational level have more difficulty understanding health information and making informed screening decisions, we hypothesize that the relationship between education and IDM involves indirect pathways via multiple domains of HL. Specifically, we aim to assess whether multiple HL domains can explain educational inequalities in IDM in CRC screening. Figure 1 presents the conceptual model that will be tested in this study. Level of education is included in the model as an independent variable, HL as a mediator and elements of decision making in CRC screening as dependent variables. We will examine multiple elements of IDM, as there is not one operationalization of IDM or “gold standard” that does justice to the way individuals make decisions in practice [10]. Using health decision-making models, such as the health belief model [17] and the theory of planned behavior [18], we study nine elements of decision making in CRC screening, including CRC knowledge, CRC screening knowledge, attitude, decisional self-efficacy, risk perception, injunctive norm (i.e., beliefs about what others approve and disapprove), descriptive norm (i.e., beliefs about what others do), decisional conflict and decisional certainty that have been shown to be determinants of screening decision making and behavior [19,20].

We hypothesize that HL mediates the pathways between educational level and nine elements of IDM about CRC screening (Figure 1). Four hypotheses will be tested for each of the four domains of health literacy:

**Hypothesis 1** **(H1):** *Comprehension mediates the pathway between educational level and informed decision making*.

**Hypothesis 2** **(H2):** *Application mediates the pathway between educational level and informed decision making*.

**Hypothesis 3** **(H3):** *Numeracy mediates the pathway between educational level and informed decision making*.

**Hypothesis 4** **(H4):** *Communication mediates the pathway between educational level and informed decision making.*

## 2. Methods

### 2.1. Study Participants

Participants (N = 696) were recruited from a large participant pool by two ISO-certified market research companies (Flycatcher and PanelClix). A stratified sample of individuals eligible for CRC screening (55–75 years of age) was created with high/middle and low educational levels equally represented. Individuals were eligible to participate in the study if they were able to read or understand Dutch and had been invited to participate in the Dutch CRC screening program in 2017 (individuals born in 1942, 1944, 1956, 1958 or 1960 received a CRC screening invitation in 2017). The market research companies sent all eligible participants an email to explain the purpose and procedure of the study and to invite them to participate in the study. 

### 2.2. Data Collection

Demographic characteristics (birth year and sex), education and health literacy were first assessed by an online questionnaire among 696 participants in January 2017 (T0). Of these 696 participants, 407 (58%) completed the second questionnaire in February 2017 (T1), and 327 (47%) completed a third questionnaire in December 2017 (T2). At T1, participants were instructed to read the online information leaflet about CRC screening from the National Institute of Public Health and the Environment (RIVM) [21]. The following variables were assessed after participants had read the leaflet: CRC knowledge, screening knowledge, attitude towards CRC screening, self-efficacy regarding decision making about CRC screening, risk perception, injunctive norm, descriptive norm, and decisional conflict. Decisional certainty was assessed by a third questionnaire (T2) in December 2017 after invitees had made the decision about whether or not to participate in CRC screening (*n* = 327).

### 2.3. Independent Variable

*Educational level* was categorized as low (levels 0–2 early childhood; primary education; lower secondary education); middle (levels 3–5: upper secondary; postsecondary; short-cycle tertiary); and high (levels 6–8; bachelor; master; doctoral), following the International Standard Classification of Education (ISCED) [22].

### 2.4. Mediators

Four domains of HL were assessed: *Comprehension*, *Application*, *Numeracy* and *Communication*. These four domains were validated in the context of IDM in CRC screening in a previous study [23]. Although a fifth domain, namely *Appraisal*, was also identified to be important for IDM, this domain did not meet acceptable psychometric properties and was therefore not examined in this study.

Comprehension was assessed by a 13 item version of the Short Assessment of Health Literacy in Dutch (SAHL-D). The SAHL-D consists of single words that refer to medical specialties, tests, treatment and symptoms. People have to select the correct meaning of each word [24]. The 13 item version of the comprehension test has previously been validated, with higher scores indicating higher *Comprehension* skills (scores range from 0 to 13) [25].

Application was assessed by the Newest Vital Sign in Dutch (NVS-D) [26], consisting of questions about an ice cream nutrition label, with higher scores indicating higher *Application* skills (scores range from 0 to 4).

Numeracy was assessed by four items of a risk scale [27], with higher scores indicating higher *Numeracy* skills (scores range from 0 to 4).

Communication was assessed by the Perceived Efficacy in Patient–Physician Interactions (PEPPI) [28], with higher scores indicating higher perceived self-efficacy in *communication* with a health care provider (scores range from 0 to 5).

### 2.5. Dependent Variables

CRC knowledge was assessed by 6 items about CRC in general [29] (example item: “Colorectal cancer has a better chance of survival when detected in an early stage”). Items were statements with response options “correct”, “incorrect” or “don’t know”. Responses that correctly identified a given statement as “correct” or “incorrect” were scored as 1; responses that incorrectly identified a statement as “correct” or “incorrect” or the use of “don’t know” were classified as 0. Summing of the scores resulted in individual knowledge scores ranging from 0 to 6, with higher scores indicating better CRC knowledge. 

CRC screening knowledge was assessed by 12 items about CRC screening [29] (example item: “If the stool test detects blood, there is a 100% chance of colorectal cancer”). All items were statements with response options “correct”, “incorrect” or “don’t know”. Responses that correctly identified a given statement as “correct” or “incorrect” were scored as 1; responses that incorrectly identified a statement as “correct” or “incorrect” or the use of “don’t know” were classified as 0. Summing of the scores resulted in individual knowledge scores ranging from 0 to 12, with higher scores indicating better CRC screening knowledge. 

Attitude towards own participation in CRC screening was assessed with 10 items using 5-point Likert scales that were used in a Dutch study on decision making in CRC screening [29]. Participants were asked to indicate whether participation in CRC screening for themselves would be a good–bad idea, frightening–not frightening, reassuring–not reassuring, self-evident–not self-evident, important–unimportant, wise–unwise, desirable–undesirable, pleasant–unpleasant, harmful–not harmful, and useful–not useful. Scores were summed, resulting in a total attitude scale ranging from 10 to 50, with higher scores indicating a more positive attitude towards CRC screening. 

Descriptive norm was measured by one item (‘People like me participate in CRC screening’) using a 5-point Likert scale (1 = totally disagree; 5 = totally agree). A higher score indicated a higher descriptive norm. 

Injunctive norm was measured by one item (‘Important others will approve of my participation in CRC screening’) using a 5-point Likert scale (1 = totally disagree; 5 = totally agree). A higher score indicated a higher injunctive norm. 

Decisional self-efficacy was measured by one item (‘I am confident that I am able to make the decision about whether or not participate myself’) using a 5-point Likert scale (1 = not at all; 5 = a lot). A higher score indicated higher decisional self-efficacy.

Risk perception was measured by summing the scores of two items (‘I think my chances of getting CRC are big’ and ‘I think that my chance of getting CRC is bigger than that of others my age’) using 5-point Likert scales (1 = totally disagree; 5 = totally agree), with scores ranging from 2 to 10 and a higher score indicating higher risk perception.

Decisional conflict was assessed with the low literacy version of the Decisional Conflict Scale (DCS) [30], which has been used in a previous Dutch study [31]. This version consists of 10 items with three response options of “yes” (scored as 0), “don’t know” (scored as 2), or “no” (scored as 4). The sum of the scores was multiplied by 2.5 following the original scoring [31], with higher scores indicating higher decisional conflict.

Decisional certainty was measured by one item (‘How certain are you that you made the right decision for yourself?’) on a 4-point Likert scale (1 = not at all; 4 = a lot). A higher score indicated lower decisional certainty.

### 2.6. Data Analysis

Descriptive analyses were performed using SPSS 24.0 and mediation analyses were conducted using Mplus version 8.0. First, we explored educational differences using known group comparisons. Taking into account multiple testing, we chose the significance level of *p*-value ≤ 0.01 (*p* = 0.05/three comparisons). Mediation analyses were performed using structural equation modeling (SEM) as recommended by McKinnon [32] and Gunzler, et al. [33]. SEM has several advantages over standard regression methods for mediation analysis: (1) it can be used to examine direct and indirect (i.e., mediation) relationships simultaneously and (2) it can be used to test multiple independent variables, mediators and outcome variables.

In order to understand the possible mediation effects of each aspect of HL individually, H1, H2, H3 and H4 were tested in four mediation models, for each of the four domains of health literacy (*Comprehension*, *Application*, *Numeracy* and *Communication*) separately. In each model, the independent variable was educational level and the dependent variables were nine separate elements of IDM: CRC knowledge, screening knowledge, attitude, injunctive norm, descriptive norm, decisional self-efficacy, risk perception, decisional conflict and decisional certainty (see Figure 1). The mediating variable was one of the HL domains. All variables were included in the models as continuous variables, with the exception of educational level, which was treated as a dummy variable [34]. For each model, three comparisons were made: low vs. middle, low vs. high and middle vs. high educational level. As we included all pathways into the model (i.e., all direct and indirect effects), the resulting models were so-called ‘just-identified’ models, meaning that the models have zero degrees of freedom and cannot be used to evaluate model fit. Bootstrapping analyses were conducted with 1000 bootstrapped samples in order to obtain reliable 95% confidence intervals of estimated parameters which were not normally distributed [35].

Hypotheses regarding the mediation effect of HL on the relation between educational level and the elements of IDM were evaluated by looking at whether zero is in the confidence interval of the associated indirect effect. If zero is not in the interval, one can be confident that the mediation effect is statistically significant [32]. For all pathways, standardized total, direct and indirect effects were reported. The total effect (c-coefficient) is the sum of the indirect effect (ab) and the direct effect (c’-coefficient) (see Figure 1). When the indirect effect of education on an element of decision making through one of the HL domains is significant, this indicates that there is at least partial mediation. If the direct effect of education on the same element of decision making is not significant, there is full mediation. Values of indirect effects of 0.02, 0.15 and 0.40 were interpreted as ‘small’, ‘medium’, or ‘large’ respectively following recommendations by Kenny [36]. Moreover, to give an indication of the extent of the mediating role of the HL domains in the relationship between educational level and decision-making outcomes, we also calculated the percentage of the total effect that was mediated by the HL domains (ab/(ab + c’ × 100%)) [37].

### 2.7. Ethical considerations

According to Dutch law, this study was waived from requiring ethical approval by the Medical Ethics Review Committee of Amsterdam UMC (location AMC), since the Dutch Medical Research Involving Human Subjects Act did not apply to the study (reference number W14_073 #14.17.0099). We ensured anonymity of the participants, informed participants about the objectives of the study, and obtained informed consent prior to participation in each online questionnaire in this study.

## 3. Results 

### 3.1. Descriptive Statistics

The final sample included 407 participants—of which, 183 (45.0%) were males and 224 (55.0%) were females. As shown in Table 1, 91 of the 407 (22.4%) participants had a low educational level, 155 (38.0%) had a middle educational level and 161 (39.6%) had a high educational level. Participants with a low educational level scored lowest on all HL measures. There were no significant educational differences in the self-reported reading of the CRC screening information before participants completed the survey questions.

### 3.2. Education and Health Literacy Domains (a-Path)

**Figure 2:**
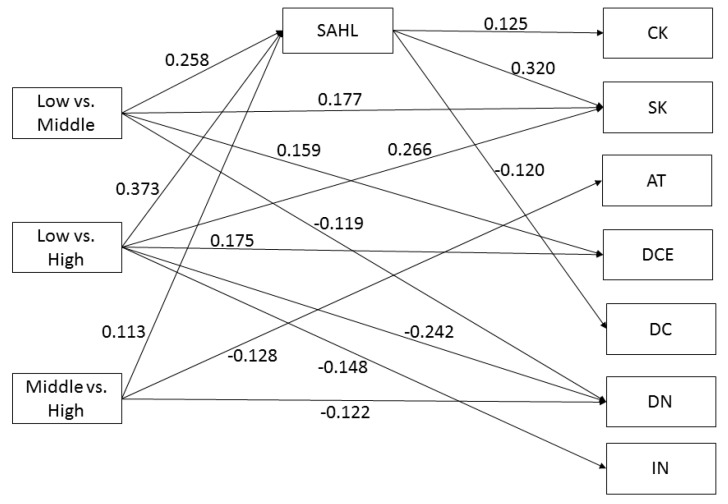
Comprehension (SAHL) as mediator. Note: CK = CRC knowledge; SK = screening knowledge; AT = attitude; DCE = decisional certainty; DC = decisional conflict; DN = descriptive norm. Non-significant paths are not shown.

**Figure 3:**
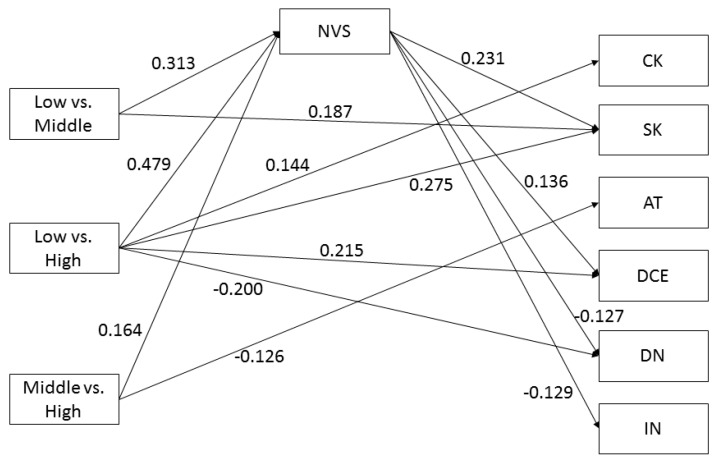
Application as mediator. Note: CK = CRC knowledge; SK = screening knowledge; AT = attitude; DCE = decisional certainty; DN = descriptive norm; IN = injunctive norm. Non-significant paths are not shown.

**Figure 4:**
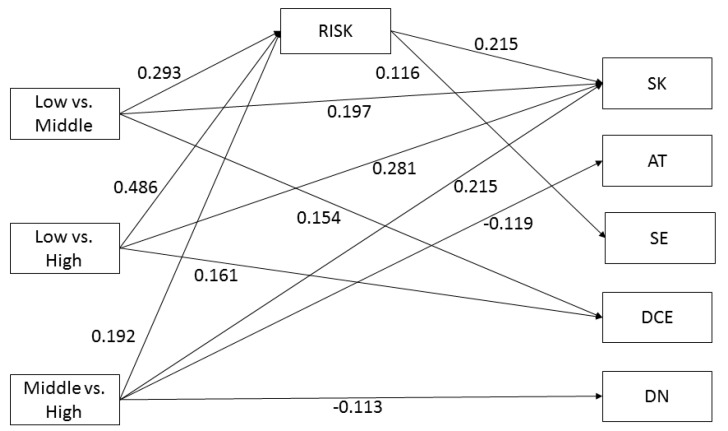
Numeracy as mediator. Note: SK = screening knowledge; AT = attitude; SE = self-efficacy; DCE = Decisional certainty; DN = Descriptive norm. Non-significant paths are not shown.

**Figure 5:**
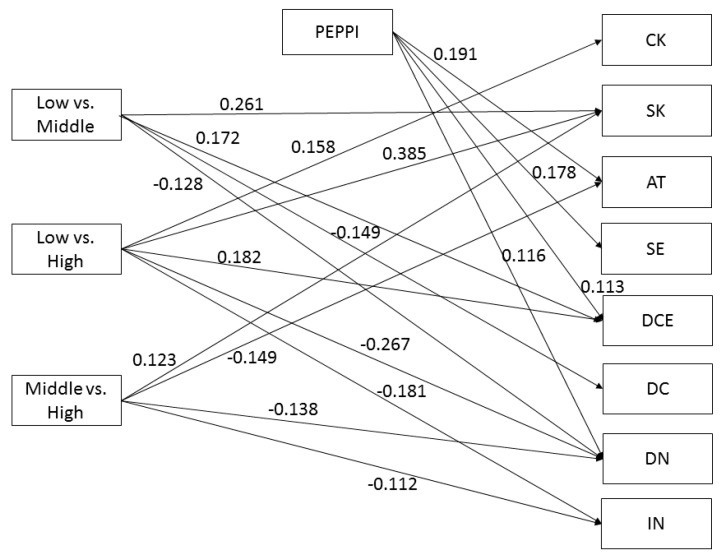
Communication as mediator. Note: CK = CRC knowledge; SK = screening knowledge; AT = attitude; SE = self-efficacy; DCE = decisional certainty; DC = decisional conflict; DN = descriptive norm; IN = injunctive norm. Non-significant paths are not shown.

A higher educational level was significantly associated with higher scores on the three HL domains *Comprehension* (low vs. middle education: B = 0.258; CI 95% [0.129; 0.388]; low vs. high education: B = 0.373; CI 95% [0.246; 0.501]; middle vs. high education: B = 0.113; CI 95% [0.013; 0.214]), *Application* (low vs. middle education: B = 0.313; CI 95% [0.188; 0.437]; low vs. high education: B = 0.479; CI 95% [0.363; 0.594]; middle vs. high education: B = 0.164; CI 95% [0.069; 0.259]), and *Numeracy* (low vs. middle education: B = 0.293; CI 95% [0.184; 0.401]; low vs. high education: B = 0.486; CI 95% [0.381; 0.592], middle vs. high education: B = 0.192; CI 95% [0.089; 0.294]), but not with higher scores on the HL domain *Communication* (see Figure 2, Figure 3, Figure 4 and Figure 5 for the standardized estimates (between −1 and 1) for the causal paths of the direct and indirect effects).

### 3.3. Health Literacy Domains and Elements of Informed Decision Making (b-Path)

Higher *Comprehension* scores, as measured by the SAHL-D, were significantly associated with higher CRC knowledge (B = 0.125; CI 95% [0.025; 0.224]), higher CRC screening knowledge (B = 0.320; CI 95% [0.227; 0.413]) and lower decisional conflict (B = −0.120; CI 95% [−0.222; −0.018]) (see Figure 2), but not with decisional self-efficacy, risk perception, injunctive norm, descriptive norm, attitude and decisional certainty (not shown).

Higher *Application* scores, as measured by the NVS, were significantly associated with higher screening knowledge (B = 0.231; CI 95% [0.125; 0.337]), lower injunctive norm (B = −0.129; CI 95% [−0.228; −0.030]), lower descriptive norm (B = −0.127; CI 95% [−0.224; −0.030]) and higher decisional certainty (B = 0.136; CI 95% [0.025; 0.248]) (see Figure 3), but not with CRC knowledge, CRC self-efficacy, risk perception, attitude, and decisional conflict (not shown).

Higher *Numeracy* scores, as measured by the risk tool, were significantly associated with higher CRC screening knowledge (B = 0.215; CI 95% [0.120; 0.310]) and higher decisional self-efficacy (B = 0.116; CI 95% [0.020; 0.213]) (Figure 4), but not with CRC knowledge, risk perception, attitude, decisional conflict and decisional certainty (not shown).

Higher *Communication* scores, as measured by the PEPPI, were significantly associated with higher decisional self-efficacy (B = 0.178; CI 95% [0.081; 0.275]), higher descriptive norm (B = 0.116; CI 95% [0.020; 0.213]), more positive attitude (B = 0.191; CI 95% [0.100; 0.281]) and higher decisional certainty (B = 0.113; CI 95% [0.002; 0.225]) (see Figure 5), but not with CRC knowledge, CRC screening knowledge, risk perception, injunctive norm and decisional conflict (not shown).

### 3.4. Education and Elements of Informed Decision Making (c-Path)

Table 2, Table 3, Table 4 and Table 5 show the total effect of education on the nine elements of IDM: CRC knowledge, screening knowledge, attitude, self-efficacy, risk perception, injunctive norm, descriptive norm, decisional conflict, and decisional certainty, adjusted for HL (c’-coefficient). Higher educational level was significantly associated with six of the nine IDM elements: higher CRC knowledge, higher CRC screening knowledge, lower injunctive norm, lower descriptive norm, lower decisional conflict and higher decisional certainty. The total effects of education on these decision-making outcomes differed for the three comparisons (low vs. middle, low vs. high and middle vs. high education). For all three comparisons, the total effects of education on CRC screening knowledge and descriptive norm were significant. In addition, for the comparisons low vs. middle and low vs. high education, the total effects of education on CRC knowledge, decisional conflict and decisional certainty were significant. For low vs. high education only, the total effect of education on injunctive norm was significant. For middle vs. high education only, the total effect of education on attitude was significant.

### 3.5. Education and Informed Decision Making Mediated through Health Literacy Domains (ab-Path)

#### 3.5.1. Mediator: Comprehension

Table 2 presents the mediation effects of *Comprehension (H1)*. The results indicate that *Comprehension* mediates the effect between education (low vs. middle and low vs. high) and CRC knowledge, the effect between education (low vs. middle, low vs. high and middle vs. high) and CRC screening knowledge, and the effect between education (low vs. middle and middle vs. high) and decisional conflict. All mediation effects of *Comprehension* were of small effect size (0.03–0.12) and explained 25–31% of the total effect.

#### 3.5.2. Mediator: Application

Table 3 presents the mediation effects of *Application (H2)*. The results indicate that *Application* mediates the effects between education and CRC Screening knowledge, Injunctive Norm, Descriptive Norm, and Decisional Certainty. These mediating effects were significant for the comparisons low vs. middle and low vs. high education. For CRC Screening knowledge only, the mediating effect was significant for all three comparisons (low vs. middle, low vs. high and middle vs. high education). The mediating effects were of small effect size (0.04–0.11), explaining 23–57% of the total effect. 

#### 3.5.3. Mediator: Numeracy

Table 4 presents the mediation effects of *Numeracy (H3)*. The results indicate that *Numeracy* mediates the effect between education (low vs. middle and low vs. high) and CRC screening knowledge (small effect sizes of 0.06 and 0.11, explaining 24–27% of the total effect) and mediates the effect between education (low vs. middle and low vs. high) and decisional self-efficacy (small effect size of 0.03, explaining 67% of the total effect).

#### 3.5.4. Mediator: Communication

We found no mediation effects of *Communication* in the relationship between educational level and the nine decision-making outcomes (H4), as the association between education and *Communication* was not significant (a-path) (Table 5).

## 4. Discussion

In this study, we have explored associations between HL and IDM in individuals eligible for CRC screening, and assessed to what extent HL mediates the association between level of education and nine elements of IDM. Informed decision making in CRC screening requires an individual to understand CRC screening information, derive meaning for their personal situation, appraise information, communicate with others, express decision-making preferences, weigh up personal pros and cons, use information, follow instructions and translate a decision into actual participation [15]. Our findings support previous research that shows that a lower HL affects key elements of informed decision making [38,39]. In addition, this study shows that three domains of HL, namely *Comprehension (H1)*, *Application (H2)* and *Numeracy (H3)*, partially mediate educational inequalities in the following elements of IDM: knowledge about CRC and CRC screening, social norms (i.e., injunctive and descriptive norm) and confidence in the decision (i.e., decisional conflict and decisional certainty).

Consistent with previous research, our study underlines that multiple domains of HL are required for IDM in CRC screening [16]. Using a HL measurement instrument that we previously validated in the context of CRC screening [23], we assessed four domains of HL: *Comprehension*, *Application*, *Numeracy* and *Communication*. Of these, the domains *Comprehension*, *Application* and *Numeracy* were found to be significantly related to all elements of informed decision making, with the exception of risk perception. In line with previous research, we found no association between HL and participants believing that their chances of getting CRC are big and bigger than others their age [14].

In line with other studies, we found that individuals with a low HL had low knowledge of CRC and CRC screening [14,31,39], a less positive attitude towards CRC screening [11,39], lower decisional self-efficacy [13] and higher decisional conflict [31]. To our knowledge, no other study has examined the association between HL and social norms about CRC screening. A recent study, however, did show that increasing social norms about CRC screening uptake increases CRC screening intention [40]. Further research should examine how social norms may influence IDM in CRC screening among those with a low HL.

Comprehension mediated the effect between education, CRC knowledge, CRC screening knowledge and decisional conflict (H1). This means that having lower *Comprehension* skills partly explains why lower educated participants are more likely to have lower CRC and screening knowledge and to experience more decisional conflict. *Application* mediated the effect between education and Screening knowledge, Injunctive Norm, Descriptive Norm and Decisional Certainty (H2). Hence, individuals with a lower educational level have more difficulty applying information to their personal situation and consequently more strongly perceive that similar others participate in screening (descriptive norm) and that their participation is approved by others (injunctive norm). *Numeracy* mediated the effect between education and Screening knowledge and decisional self-efficacy (H3), suggesting that having lower numeracy skills partly explains why being lower educated is associated with having less knowledge of CRC screening and less self-efficacy regarding the decision to participate in screening.

The mediating effects of the HL domains were generally of small effect size and explained between 23% and 7% of the total effects. In terms of the percentage of the total effect explained, the mediation of *Application* in the association between education and injunctive norm (explaining 60%), and the mediation of *Numeracy* in the association between education and decisional self-efficacy (explaining 70%) were most notable. In line with previous research on the relationship between education, health literacy and health outcomes [41], our findings show that HL plays a larger role among those with a lower education than those with higher education for most of the IDM outcomes.

Of note, we found no mediating effects of *Communication (H4)* as the association between education and *Communication* was not significant. The latter might be explained by the use of a self-report measure to assess *Communication* skills [29], rather than assessing actual communication skills. Individuals with a low educational level reported having a slightly higher (albeit not significant) confidence in their communication skills as compared to those with a middle educational level, which might imply an overestimation of *Communication* skills. Performance-based HL measures have shown to be better at discriminating between different levels of educational levels than self-report measures [26].

A recent review showed that most HL interventions in the European Union (EU) focus on either functional HL or numeracy [42]. Our findings suggest that interventions to decrease educational inequalities in informed decision in the context of IDM in CRC screening should focus on multiple HL domains to mitigate the effects of “uninformed” decision making. This means that decision-support interventions, such as (digital) communication and decision-support tools, should not only support reading and understanding of (numerical) CRC screening information, but should also support weighing up potential harms and benefits of CRC screening and clarifying personal values (i.e., attitudes) to increase knowledge of CRC and CRC screening, increase decisional self-efficacy, correct social norms, reduce decisional conflict and increase decisional certainty. As HL does not explain all the variance in the relation between education and IDM, having adequate HL by itself may not be sufficient for IDM in CRC screening. The determinants of IDM are numerous and complex, and encompass influences from the individual, the family, the provider and the health care system. Further research should explore factors other than HL that may also play a role in this association.

In the current study, we used multiple elements of IDM in CRC screening as there is no “gold standard” measure of IDM [10]. However, in order to understand how to facilitate IDM in CRC screening, it is crucial to first specify and operationalize a definition of IDM that is in line with how individuals make decisions in daily practice. The lack of a valid definition of IDM hampers generalization of findings to other situations. Common definitions of IDM, such as the one by Marteau, et al. [43], who describe an informed decision as one that is based on relevant knowledge, consistent with one’s personal values, and is behaviorally implemented, are based on a rational decision model, assuming that individuals deliberately weigh up pros and cos of CRC screening, and thereby fail to recognize other factors that play a role in decision making. To truly understand the role of HL in IDM, and for the development of and evaluation of decision-support interventions, a new definition and universal measure of IDM should be developed.

The main strength of the current study is that we included multiple aspects of HL as well as IDM in our model and measurements. In addition, the use of longitudinal data allowed us to examine whether HL precedes the elements of IDM, presenting a more accurate representation of the temporal order, leading to more accurate conclusions about mediation [33]. This study also has important limitations. First, although we identified five HL domains as important in our previous study [23], the HL domain *Appraisal* did not meet the psychometric properties and has therefore not been examined in the present study, leaving an important pathway unexplored. Second, we were not able to demonstrate an association between education and *Communication*, which can be explained by the use of a self-report measure. To obtain a better understanding of the association between competency in communicating with health care professionals and decision-making outcomes, more research is needed for the development of performance-based measures for this domain. Third, we included nine elements for IDM, but decision making is a complex process which involves many other variables that have not been taken into account in this study. Lastly, the generalizability of our results is limited, as our study was conducted among individuals eligible for the Dutch CRC screening program. Although caution is needed in drawing conclusions, we believe that the HL skills needed for IDM in CRC screening can to a large extent be generalized to other European population-based CRC screening programs, as the organization of these programs show similarities to the Dutch CRC screening program [1].

## 5. Conclusions

By involving multiple elements of IDM, this study supports our thesis that multiple domains of HL are important in the context of informed decision making in CRC screening. Our results suggest that the HL domains *Comprehension*, *Application* and *Numeracy* mediate the association between education and elements of IDM in CRC screening. Hence, decision-support interventions for reducing educational inequalities in IDM should be tailored to multiple HL domains. For example, adapting CRC screening information in a way that can be more readily accessed, understood and used by those who have difficulty reading, understanding and appraising CRC screening information may facilitate IDM in CRC screening.

## Figures and Tables

**Table 1:** Participant characteristics (*n* = 407) per educational level (low, middle, and high).

Participant Characteristics	Educational Level			
	Low (*n* = 91) % or Mean (SD)	Middle (*n* = 155) % or Mean (SD)	High (*n* = 161) % or Mean (SD)	All % or Mean (SD)
Gender				
Male	29 (31.9)	76 (49.0)	78 (48.4)	183 (45.0)
Female	62 (68.1)	79 (51.0)	83 (51.6)	224 (55.0)
Read the CRC Screening Information				
Yes/A bit	82 (90.1)	146 (94.2)	157 (97.5)	385 (94.6)
No	9 (9.9)	9 (5.8)	4 (2.5)	22 (5.4)
Health Literacy Domain/Scale				
Comprehension/SAHL-D	7.84 (3.28) ^a,b^	9.46 (2.84) ^b,c^	10.17 (2.84) ^a,c^	9.38 (3.07)
Numeracy/Risk	1.43 (1.07) ^a,b^	2.2 (1.21) ^b,c^	2.70 (1.23) ^a,c^	2.23 (1.28)
Application/NVS-D	3.58 (1.68) ^a,b^	4.61 (1.56) ^b,c^	5.15 (1.30) ^a,c^	4.59 (1.60)
Communication/PEPPI	20.27 (2.26)	20.04 (2.78)	20.52 (2.92)	20.28 (2.73)
IDM Elements				
CRC Knowledge	4.22 (1.13)	4.50 (1.03)	4.55 (0.98)	4.46 (1.04)
Screening Knowledge	8.05 (2.09) ^a,b^	9.03 (1.75) ^b,c^	9.50 (1.52) ^a,c^	9.00 (1.83)
Attitude	41.38 (7.14)	41.94 (6.66) ^c^	40.0 (7.59) ^c^	41.05 (7.18)
Decisional Self-efficacy Missing	4.40 (0.75) 1 (1.1)	4.48 (0.68) 4 (2.6)	4.59 (0.63) 1 (0.6)	4.50 (0.68) 6 (1.5)
Risk Perception Missing	5.67 (1.25) 1 (1.1)	5.67 (1.17) 4 (2.6)	5.63 (1.00) 1 (0.6)	5.65 (1.12) 6 (1.5)
Injunctive Norm Missing	3.39 (1.43) ^a^ 1 (1.1)	3.19 (1.30) 2 (1.3)	2.89 (1.34) ^a^ 0	3.11 (1.35) 3 (0.7)
Descriptive Norm Missing	4.24 (0.88) ^a^ 1 (1.1)	3.95 (1.00) 2 (1.3)	3.68 (1.31) ^a^ 0	3.91 (1.05) 3 (0.7)
Decisional Conflict Missing	31.33 (22.74) 1 (1.1)	25.20 (19.38) 5	24.72 (20.03) 1 (0.6)	26.39 (20.56) 7 (1.72)
Decisional Certainty Missing	9.44 (1.55) ^b^ 20 (22.0)	9.93 (1.45) ^b^ 25 (16.1)	10.00 (1.49) 35 (21.7)	9.85 (1.50) 80 (19.7)

^a^ Significant differences between participants with a low and high educational level (*p* < 0.01). ^b^ Significant differences between participants with a low and middle educational level (*p* < 0.01). ^c^ Significant differences between participants with a middle and high educational level (*p* < 0.01). CRC = colorectal cancer; SAHL-D = Short Assessment of Health Literacy in Dutch; NVS-D = Newest Vital Sign in Dutch; PEPPI = Perceived Efficacy in Patient–Physician Interactions.

**Table 2:** Standardized total, direct and total indirect effects: mediator *Comprehension*.

Comparison	Effect	CK	SK	AT	SE	RP	IN	DN	DC	DCE
Low * vs. Middle Education	Total	0.129 *	0.260 *	0.038	0.051	0.002	−0.072	−0.133 *	−0.147 *	0.168 *
	Direct	0.097	0.177 *	0.047	0.034	0.007	−0.050	−0.119 *	−0.116	0.159 *
	Indirect	0.032 *	0.083 *	−0.009	0.016	−0.005	−0.022	−0.014	−0.031	0.009
	Effect size	**0.25**	**0.32**	−0.24	0.31	−2.5	0.31	0.11	0.21	0.05
Low * vs. High Education	Total	0.156*	0.386 *	−0.094	0.087	−0.018	−0.179 *	−0.262 *	−0.157 *	0.187 *
	Direct	0.110	0.266 *	−0.081	0.063	−0.011	−0.148 *	−0.262 *	−0.112	0.175 *
	Indirect	0.046 *	0.119 *	−0.014	0.024	−0.007	−0.031	−0.020	−0.045 *	0.012
	Effect size	**0.29**	**0.31**	0.15	0.28	0.39	0.17	0.08	**0.29**	0.06
Middle * vs. High Education	Total	0.026	0.124 *	−0.132 *	0.036	−0.020	−0.107 *	−0.128 *	−0.009	0.018
	Direct	0.012	0.087	−0.128 *	0.029	−0.018	−0.098	−0.122 *	0.004	0.014
	Indirect	0.014	0.038 *	−0.004	0.007	−0.002	−0.010	−0.006	−0.014	0.004
	Effect size	0.54	**0.31**	0.03	0.19	0.10	0.09	0.05	1.56	0.22

* = Reference category. Note: CK = CRC knowledge; SK = screening knowledge; AT = attitude; SE = self-efficacy; RP = risk perception, IN = injunctive norm; DN = descriptive norm; DC = decisional Conflict; DCE = decisional certainty. Note. Significant results printed in bold. The total effects for SE, RP, IN, DN and DCE slightly differ because of missing values.

**Table 3:** Standardized total, direct and total indirect: mediator *Application*.

Comparison	Effect	CK	SK	AT	SE	RP	IN	DN	DC	DCE
Low * vs. Middle Education	Total	0.129 *	0.260 *	0.038	0.058	0.002	−0.070	−0.132 *	−0.147 *	0.175 *
	Direct	0.121	0.187 *	0.049	0.026	0.001	−0.030	−0.092	−0.133 *	0.132
	Indirect	0.017	0.072 *	−0.012	0.033	0.001	−0.040 *	−0.040 *	−0.013	0.043 *
	Effect size	0.13	**0.28**	−0.31	0.57	0.5	**0.57**	**0.30**	0.09	**0.25**
Low * vs. High Education	Total	0.156 *	0.386 *	−0.094	0.133	−0.018	−0.178 *	−0.261 *	−0.157 *	0.193 *
	Direct	0.144 *	0.275 *	−0.077	0.083	−0.020	−0.116 *	−0.200 *	−0.137	0.127
	Indirect	0.012	0.111 *	−0.018	0.050	0.002	−0.062 *	−0.061 *	−0.020	0.065 *
	Effect size	0.08	**0.29**	0.19	0.38	−0.11	**0.35**	**0.23**	0.13	**0.34**
Middle * vs. High Education	Total	0.026	0.124 *	−0.132 *	0.074	−0.020	−0.107 *	−0.128 *	−0.009	0.018
	Direct	0.022	0.087	−0.126 *	0.057	−0.021	−0.086	−0.107	−0.002	0.014
	Indirect	0.004	0.038 *	−0.006	0.017	0.001	−0.021	−0.021	−0.007	0.004
	Effect size	0.15	**0.31**	0.04	0.23	−0.05	0.20	0.16	0.78	0.22

* = Reference category. Note: CK = CRC knowledge; SK = screening knowledge; AT = attitude; SE = self-efficacy; RP = risk perception, IN = injunctive norm; DN = descriptive norm; DC = decisional conflict; DCE = decisional certainty. Note. Significant results printed in bold. The total effects for SE, RP, IN, DN and DCE slightly differ because of missing values.

**Table 4:** Standardized total, direct and total indirect: mediator *Numeracy*.

Comparison	Effect	CK	SK	AT	SE	RP	IN	DN	DC	DCE
Low * vs. Middle Education	Total	0.129 *	0.260 *	0.038	0.051	0.002	−0.071	−0.133 *	−0.147 *	0.170 *
	Direct	0.117	0.197 *	0.058	0.017	−0.007	−0.045	−0.109	−0.130	0.154
	Indirect	0.012	0.063 *	−0.020	0.034 *	0.009	−0.026	−0.023	−0.016	0.016
	Effect size	0.09	**0.24**	−0.53	**0.67**		0.37	0.17	0.11	0.09
Low * vs. High Education	Total	0.156 *	0.386 *	−0.094	0.087	−0.018	−0.179 *	−0.262 *	−0.157 *	0.188 *
	Direct	0.136	0.281 *	−0.061	0.030	−0.033	−0.135	−0.223 *	−0.130	0.161 *
	Indirect	0.020	0.105 *	−0.034	0.057 *	0.015	−0.044	−0.039	−0.027	0.026
	Effect size	0.128	**0.27**	0.36	**0.66**	−0.83	0.25	0.15	0.17	0.14
Middle * vs. High Education	Total	0.026	0.124 *	−0.132 *	0.035	−0.020	−0.108 *	−0.129 *	−0.009	0.017
	Direct	0.018	0.041 *	−0.119 *	0.013	−0.026	−0.090	−0.113 *	0.001	0.006
	Indirect	0.008	0.083	−0.013	0.022	0.006	−0.017	−0.015	−0.011	0.010
	Effect size	0.31	0.67	0.10	0.63	−0.3	0.16	0.12	1.22	0.59

* = Reference category. Note: CK = CRC knowledge; SK = screening knowledge; AT = attitude; SE = self-efficacy; RP = risk perception, IN = injunctive norm; DN = descriptive norm; DC = decisional conflict; DCE = decisional certainty. Note. Significant results printed in bold. The total effects for SE, RP, IN, DN and DCE slightly differ because of missing values.

**Table 5:** Standardized total, direct and total indirect: mediator *Communication*.

Comparison	Effect	CK	SK	AT	SE	RP	IN	DN	DC	DCE
Low * vs. Middle Education	Total	0.129 *	0.260 *	0.038	0.059	0.002	−0.071	−0.133 *	−0.147 *	0.167 *
	Direct	0.127	0.261 *	0.046	0.066	0.004	−0.002	−0.128	−0.149 *	0.172 *
	Indirect	0.002	−0.001	−0.008	−0.007	−0.003	−0.069	−0.005	0.002	−0.005
	Effect size	-	-	-	-	-	-	-	-	-
Low * vs. High Education	Total	0.156 *	0.386 *	−0.094	0.134	−0.018	−0.179 *	−0.262 *	−0.157 *	0.186 *
	Direct	0.158 *	0.385 *	−0.103	0.126	−0.021	−0.181 *	−0.267 *	−0.155 *	0.182 *
	Indirect	−0.002	0.001	0.008	0.008	0.003	0.002	0.005	−0.002	0.005
	Effect size	-	-	-	-	-	-	-	-	-

* = Reference category. Note: CK = CRC knowledge; SK = screening knowledge; AT = attitude; SE = self-efficacy; RP = risk perception, IN = injunctive norm; DN = descriptive norm; DC = decisional conflict; DCE = decisional certainty. Note. Significant results printed in bold. The total effects for SE, RP, IN, DN and DCE slightly differ because of missing values.

## References

[1] von Karsa L., Patnick J., Segnan N., Atkin W., Halloran S., Lansdorp-Vogelaar I., Malila N., Minozzi S., Moss S., European Colorectal Cancer Screening Guidelines Working Group (2013). European guidelines for quality assurance in colorectal cancer screening and diagnosis: Overview and introduction to the full supplement publication. Endoscopy.

[2] Hofmann B. (2017). Ethical issues with colorectal cancer screening-a systematic review. J. Eval. Clin. Pract..

[3] Hasnain-Wynia R., Wolf M.S. (2010). Promoting health care equity: Is health literacy a missing link?. Health Serv. Res..

[4] Rimer B.K., Briss P.A., Zeller P.K., Chan E.C.Y., Woolf S.H. (2004). Informed decision making: What is its role in cancer screening?. Cancer Interdiscip. Int. J. Am. Cancer Soc..

[5] Dharni N., Armstrong D., Chung-Faye G., Wright A.J. (2017). Factors influencing participation in colorectal cancer screening—a qualitative study in an ethnic and socio-economically diverse inner city population. Health Expect..

[6] McCaffery K., Wardle J., Waller J. (2003). Knowledge, attitudes, and behavioral intentions in relation to the early detection of colorectal cancer in the United Kingdom. Prev. Med..

[7] Sørensen K., Van den Broucke S., Fullam J., Doyle G., Pelikan J., Slonska Z., Brand H. (2012). Health literacy and public health: A systematic review and integration of definitions and models. BMC Public Health.

[8] (1999). Health literacy: Report of the Council on Scientific Affairs. Ad Hoc Committee on Health Literacy for the Council on Scientific Affairs, American Medical Association. JAMA.

[9] Chesser A.K., Keene Woods N., Smothers K., Rogers N. (2016). Health Literacy and Older Adults: A Systematic Review. Gerontol. Geriatr. Med..

[10] Van der Heide I., Uiters E., Jantine Schuit A., Rademakers J., Fransen M. (2015). Health literacy and informed decision making regarding colorectal cancer screening: A systematic review. Eur. J. Public Health.

[11] Arnold C.L., Rademaker A., Bailey S.C., Esparza J.M., Reynolds C., Liu D., Platt D., Davis T.C. (2012). Literacy barriers to colorectal cancer screening in community clinics. J. Health Commun..

[12] Mitsutake S., Shibata A., Ishii K., Oka K. (2012). Association of eHealth literacy with colorectal cancer knowledge and screening practice among internet users in Japan. J. Med. Internet Res..

[13] Von Wagner C., Semmler C., Good A., Wardle J. (2009). Health literacy and self-efficacy for participating in colorectal cancer screening: The role of information processing. Patient Educ. Couns..

[14] Peterson N.B., Dwyer K.A., Mulvaney S.A., Dietrich M.S., Rothman R.L. (2007). The influence of health literacy on colorectal cancer screening knowledge, beliefs and behavior. J. Natl. Med. Assoc..

[15] Woudstra A.J., Timmermans D.R.M., Uiters E., Dekker E., Smets E.M.A., Fransen M.P. (2018). Health literacy skills for informed decision making in colorectal cancer screening: Perceptions of screening invitees and experts. Health Expect..

[16] Smith S.K., Nutbeam D., McCaffery K.J. (2013). Insights into the concept and measurement of health literacy from a study of shared decision-making in a low literacy population. J. Health Psychol..

[17] Janz N.K., Becker M.H. (1984). The Health Belief Model: A Decade Later.

[18] Ajzen I. (1991). The Theory of Planned Behavior. Organ. Behav. Hum. Decis. Process..

[19] Rawl S., Champion V., Menon U., Loehrer P.J., Vance G.H., Skinner C.S. (2001). Validation of Scales to Measure Benefits of and Barriers to Colorectal Cancer Screening. J. Psychosoc. Oncol..

[20] Sieverding M., Decker S., Zimmermann F. (2010). Information About Low Participation in Cancer Screening Demotivates Other People. Psychol. Sci..

[21] RIVM. Bevolkingsonderzoek Darmkanker [Colorectal Cancer Screening Programme]. https://www.rivm.nl/bevolkingsonderzoek-darmkanker.

[22] UNESCO Instiute for Statistics (2014). International Standard Classification of Education.

[23] Woudstra A.J., Smets E.M.A., Galenkamp H., Fransen M.P. (2019). Validation of health literacy domains for informed decision making about colorectal cancer screening using classical test theory and item response theory. Patient Educ. Couns..

[24] Pander Maat H., Essink-Bot M.L., Leenaars K.E., Fransen M.P. (2014). A short assessment of health literacy (SAHL) in the Netherlands. BMC Public Health.

[25] Woudstra A.J., Meppelink C.S., Pander Maat H., Oosterhaven J., Fransen M.P., Dima A.L. (2019). Validation of the short assessment of health literacy (SAHL-D) and short-form development: Rasch analysis. BMC Med. Res. Methodol..

[26] Fransen M.P., Leenaars K.E., Rowlands G., Weiss B.D., Maat H.P., Essink-Bot M.L. (2014). International application of health literacy measures: Adaptation and validation of the newest vital sign in The Netherlands. Patient Educ. Couns..

[27] Lipkus I.M., Samsa G., Rimer B.K. (2001). General performance on a numeracy scale among highly educated samples. Med. Decis. Mak. Int. J. Soc. Med Decis. Mak..

[28] Ten Klooster P.M., Oostveen J.C., Zandbelt L.C., Taal E., Drossaert C.H., Harmsen E.J., van de Laar M.A. (2012). Further validation of the 5-item Perceived Efficacy in Patient-Physician Interactions (PEPPI-5) scale in patients with osteoarthritis. Patient Educ. Couns..

[29] Denters M.J., Deutekom M., Essink-Bot M.-L., Bossuyt P.M., Fockens P., Dekker E. (2015). Assessing knowledge and attitudes towards screening among users of Faecal Immunochemical Test (FIT). Health Expect. Int. J. Public Particip. Health Care Health Policy.

[30] O’Connor A.M. (1995). Validation of a decisional conflict scale. Med. Decis. Mak..

[31] Essink-Bot M.L., Dekker E., Timmermans D.R.M., Uiters E., Fransen M.P. (2016). Knowledge and Informed Decision-Making about Population-Based Colorectal Cancer Screening Participation in Groups with Low and Adequate Health Literacy. Gastroenterol. Res. Pract..

[32] McKinnon D.P. (2008). Introduction to Statistical Mediation Analysis.

[33] Gunzler D., Chen T., Wu P., Zhang H. (2013). Introduction to mediation analysis with structural equation modeling. Shanghai Arch. Psychiatry.

[34] Hayes A.F., Preacher K.J. (2014). Statistical mediation analysis with a multicategorical independent variable. Br. J. Math. Stat. Psychol..

[35] MacKinnon D.P., Fairchild A.J., Fritz M.S. (2007). Mediation analysis. Annu. Rev. Psychol..

[36] Kenny D.A. Mediation. http://davidakenny.net/cm/mediate.htm.

[37] Wen Z., Fan X. (2015). Monotonicity of effect sizes: Questioning kappa-squared as mediation effect size measure. Psychol. Methods.

[38] McCaffery K.J., Holmes-Rovner M., Smith S.K., Rovner D., Nutbeam D., Clayman M.L., Kelly-Blake K., Wolf M.S., Sheridan S.L. (2013). Addressing health literacy in patient decision aids. BMC Med. Inf. Decis. Mak..

[39] Gabel P., Larsen M.B., Edwards A., Kirkegaard P., Andersen B. (2019). Knowledge, attitudes, and worries among different health literacy groups before receiving first invitation to colorectal cancer screening: Cross-sectional study. Prev. Med. Rep..

[40] Von Wagner C., Hirst Y., Waller J., Ghanouni A., McGregor L.M., Kerrison R.S., Verstraete W., Vlaev I., Sieverding M., Stoffel S.T. (2019). The impact of descriptive norms on motivation to participate in cancer screening–Evidence from online experiments. Patient Educ. Couns..

[41] Van der Heide I., Wang J., Droomers M., Spreeuwenberg P., Rademakers J., Uiters E. (2013). The Relationship Between Health, Education, and Health Literacy: Results From the Dutch Adult Literacy and Life Skills Survey. J. Health Commun..

[42] Visscher B.B., Steunenberg B., Heijmans M., Hofstede J.M., Devillé W., van der Heide I., Rademakers J. (2018). Evidence on the effectiveness of health literacy interventions in the EU: A systematic review. BMC Public Health.

[43] Marteau T.M., Dormandy E., Michie S. (2001). A measure of informed choice. Health Expect. Int. J. Public Particip. Health Care Health Policy.

